# Expression of Melatonin Synthesizing Enzymes in *Helicobacter pylori* Infected Gastric Mucosa

**DOI:** 10.1155/2013/845032

**Published:** 2013-07-10

**Authors:** Cezary Chojnacki, Tomasz Popławski, Janusz Blasiak, Jan Chojnacki, Russel J. Reiter, Grazyna Klupinska

**Affiliations:** ^1^Department of Gastroenterology, Medical University of Lodz, 1 Haller's Square, 90-647 Lodz, Poland; ^2^Department of Molecular Genetics, Faculty of Biology and Environmental Protection, University of Lodz, Pomorska 141/143, 90-236 Lodz, Poland; ^3^Department of Cellular and Structural Biology, University of Texas Health Science Center, San Antonio, TX 78229-3900, USA; ^4^Department of Clinical Nutrition, Medical University of Lodz, 1 Haller's Square, 90-647 Lodz, Poland

## Abstract

*Helicobacter pylori* colonization of gastric mucosa causes pain of unknown etiology in about 15–20% of infected subjects. The aim of the present work was to determine the level of expression of enzymes involved in the synthesis of melatonin in gastric mucosa of asymptomatic and symptomatic *H. pylori* infected patients. To diagnose *H. pylori* infection, histological analysis and the urea breath test (UBT C13) were performed. The levels of mRNA expression of arylalkylamine-N-acetyltransferase (AA-NAT) and acetylserotonin methyltransferase (ASMT) were estimated in gastric mucosa with RT-PCR. The level of AA-NAT expression and AMST was decreased in *H. pylori* infected patients and was increased after *H. pylori* eradication. We conclude that decreased expression of melatonin synthesizing enzymes, AA-NAT and ASMT, in patients with symptomatic *H. pylori* infection returns to normal level after *H. pylori* eradication.

## 1. Introduction

Melatonin is a molecule with numerous beneficial properties which is produced both in the pineal gland and in the gastrointestinal tract [1, 2]. Melatonin synthesizing enzymes are found also in other organs of mammals [3–6]. This indoleamine is produced from L-tryptophan in a metabolic pathway shared with serotonin. L-tryptophan undergoes enzymatic hydroxylation and decarboxylation to form serotonin, which is then acetylated to N-acetylserotonin by arylalkylamine N-acetyltransferase (AA-NAT). N-acetylserotonin is finally converted to N-acetyl-5-methoxytryptamine (melatonin) by acetylserotonin methyltransferase (ASMT) [7, 8]. 

Expression of AA-NAT and ASMT is regulated by adrenergic nervous system and may change under the influence of many agents, including inflammatory and hormonal factors [8, 9]. Some proinflammatory cytokines were reported to inhibit melatonin synthesis [10]. In the gut, the concentration of melatonin may also depend on the number of enterochromaffin cells (EC), which are the main source of melatonin in the gastrointestinal tract [2]. An increased number of EC cells and an elevated ASMT expression were found in inflammatory bowel disease [11–14]. On the other hand, reductions in AA-NAT and ASMT expression were observed under similar experimental conditions [15]. This apparent discrepancy may be due to nature and severity of inflammatory changes [8, 16]. 

We have found no report on the expression of AA-NAT and ASMT in gastric mucosa. However, it was observed that the number of EC cells increased in the antral part of the stomach in *H. pylori *infected patients, but it was not clear whether these changes were consequences of alterations in the expression of enzymes of the melatonin metabolic pathway or whether they influenced the clinical symptoms of gastritis [17]. The aim of the present study was to evaluate the expression of AA-NAT and ASMT in the gastric mucosa of patients with asymptomatic and symptomatic *H. pylori *infection.

## 2. Materials and Methods

### 2.1. Patients

Ninety subjects were enrolled in this study, 51 women and 39 men, aged 20–46 years (mean age 31.4 years). Clinical characteristic of the patients are presented in Table 1. The subjects were divided into three groups of 30 individuals each: I—healthy volunteers; II—subjects with asymptomatic infection of *H. pylori*; III—*H. pylori*-infected patients with dyspeptic symptoms, mainly fasting and nocturnal epigastric pain including epigastric pain syndrome, according to Rome III criteria. To diagnose *H. pylori* infection, the histological method with the Giemsa technique and the urease breath test UBT-13C on a FANCI-2 system (Fisher Instruments, Germany) were performed. Symptoms of minimum 6-month duration and no improvement after antacid or prokinetic drugs were inclusion criteria. Patients with organic, metabolic, and psychic diseases as well as individuals with long-standing pharmacological treatment and cigarette smokers were excluded from the study.

### 2.2. Study Design and Procedures

Endoscopy of the upper part of gastrointestinal tract, gastric mucosa histopathology, abdominal ultrasonography, laboratory tests, including blood cell count, CRP concentration, glucose, electrolytes, bilirubin, urea, creatinine, cholesterol, triglycerides, thyroid hormones, and the activity of aspartate aminotransferase (AST), alanine aminotransferase (ALT), gamma-glutamyl transpeptidase (GGT), alkaline phosphatase (ALP), amylase, and lipase were performed in all the subjects enrolled in the study. Seven days prior to the evaluations, all medications were withdrawn and the same diet was used by all subjects with similar daily amount of products rich in L-tryptophan.

Patients with a symptomatic *H. pylori* infection were treated with pantoprazol (40 mg), amoxicillin (1000 mg), and levofloxacin (500 mg) twice daily for 14 days, and changes in gastric symptoms were registered. Eradication of *H. pylori* was confirmed by the UBT-13C test. Control endoscopy with biopsy of the same part of stomach was performed after 3 months. Patients with asymptomatic *H. pylori* infection were not treated with any antibiotic, according to the Maastricht IV consensus [18].

Material for histological and molecular examinations was collected from the antral part (4 bioptates) and the upper part of gastric body (4 bioptates). The level of mRNA was estimated with RT-PCR and qPCR and, for this purpose, 50 mg of gastric tissue was used. Gastric tissue was excised and placed immediately in RNAlater reagent (Qiagen, Valencia, CA, USA) according to the manufacturer's instructions. For processing, samples were removed from RNAlater and RNA were isolated with Trizol (Gibco, Darmstadt, Germany) reagents with Tissue Ruptor (Qiagen). RNA pellet was reconstituted in 100 *μ*L of RNase- and DNase-free water and processed with the RNEasy Mini Kit (Qiagen) according to the manufacturer's RNA Cleanup protocol with DNase I treatment (Qiagen). Purified RNA samples were stored at −20°C until use. The quantity and quality of RNA was evaluated spectrophotometrically. cDNA synthesis was performed with oligo(dT) using ImProm-II Reverse Transcription System (Promega, Madison, WI, USA) according to the manufacturer's instruction. qPCR was conducted with PCR Master Mix (Promega) and specific primers to amplify fragments of the cDNA of the AA-NAT and ASMT genes. The qPCR products were separated on an 8% polyacrylamide gel stained with ethidium bromide. The products were then subjected to densitometry to determine the level of analysed genes mRNA. Expression of the housekeeping hypoxanthine phosphoribosyltransferase (HPRT) gene was used to normalize the level of the AA-NAT and ASMT expression. 

### 2.3. Ethics

The study was performed in accordance with the Declaration of Helsinki and with the principles of Good Clinical Practice. Written consent was obtained from each subject enrolled into the study and the study, protocol was approved by the Bioethics Committee of the Medical University in Lodz (RNN/596/11/KB).

### 2.4. Statistical Analysis

The nonparametric Kruskal-Wallis test was used for the comparison of AA-NAT and ASMT expression levels. The Mann-Whitney *U* test was applied for median comparison. The correlation between the previous parameters and intensity of *H. pylori* infection was assessed by the Pearson's correlation coefficient and linear regression equation. 

## 3. Results and Discussion

**Figure 1:**
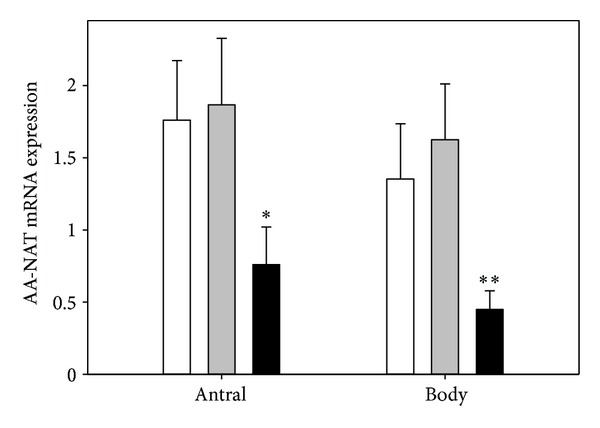
Mean relative level of arylalkylamine-N-acetyltransferase (AA-NAT) expression in antral and body gastric mucosa in healthy subjects (control, white bars) and subjects with asymptomatic (grey bars) and symptomatic (black bars) *H. pylori* infections as measured by real-time PCR. Error bars denote SD; *n* = 30 for each group; *P < 0.05, **P < 0.01 compared with the control.

Eradication is the primary treatment of *H. pylori *infection, independently whether it is associated with other symptoms. All dyspeptic patients became symptoms free after *H. pylori* eradication. However, only 4 of them (13.3%) displayed a normal histological picture of gastric mucosa. The remaining 26 patients have been scheduled for clinical, endoscopic, and histological examinations in 12 months. In healthy subjects (control, group I), AA-NAT expression in the antral mucosa was 1.76 ± 0.41, and in the body of the stomach this value was 1.35 ± 0.38 (Figure 1). In subjects with asymptomatic infection of *H. pylori* (group II), AA-NAT expression in the same parts of stomach was 1.87 ± 0.46 and 1.63 ± 0.39 (*P* > 0.05), respectively. The expression of this enzyme in dyspeptic patients with *H. pylori* infection (group III) was lower: −0.76 ± 0.26 (*P* < 0.001) and 0.45 ± 0.13 (*P* < 0.001), respectively. 

**Figure 2:**
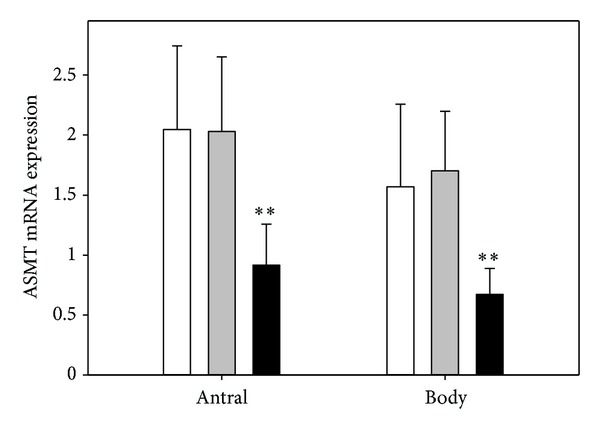
Mean relative level of acetylserotonin methyltransferase (ASMT) expression in antral and body gastric mucosa in healthy subjects (control, white bars) and subjects with asymptomatic (grey bars) and symptomatic (black bars) *H. pylori* infections as measured by real-time PCR. Error bars denote SD; *n* = 30 for each group; **P < 0.01 compared with the control.

The level of ASMT expression in antral mucosa was 2.05 ± 0.70 in group I, 2.03 ± 0.62 in group II, and 0.92 ± 0.34 (*P* < 0.001) in group III. The expression of ASMT in gastric body was 1.57 ± 0.69, 1.70 ± 0.50, and 0.67 ± 0.22 (*P* < 0.001) (Figure 2), respectively.

**Figure 3:**
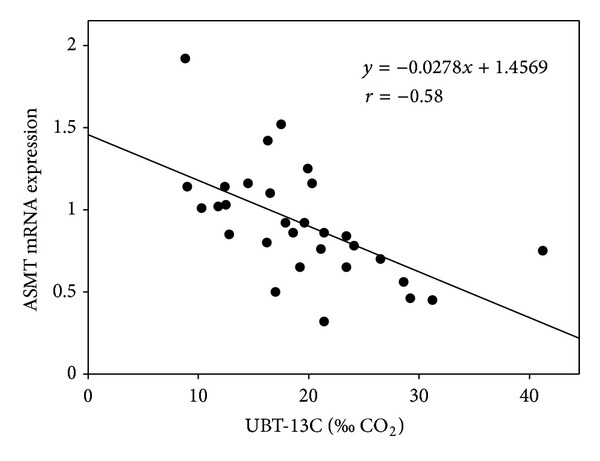
Dependence of the intensity of *H. pylori *infection intensity, measured with the urease breath test UBT-13C, on the expression of acetylserotonin methyltransferase (ASMT) in antral mucosa of patients with symptomatic infection (*n* = 30), measured by real-time PCR. Regression line was calculated by the means of the least square method.

A significant correlation between the intensity of *H. pylori* infection and the level of ASMT expression in the antral mucosa was found (*r* = −0.58, *P* < 0.01; Figure 3) in patients with symptomatic infection; no correlation in the mucosa of the stomach was observed (*r* = −0.0574, *P* > 0.05). No correlation was found in patients with asymptomatic infection. No correlation between the intensity of the infection and AA-NAT expression in both parts of the stomach was determined.

**Figure 4:**
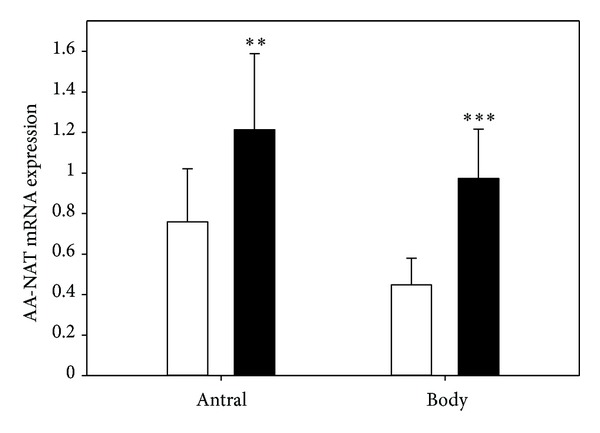
Mean relative level of arylalkylamine-N-acetyltransferase (AA-NAT) expression, measured by real-time PCR, in antral and body gastric mucosa in patients with symptomatic *H. pylori *infection before (control, white bars) and after (black bars) eradication of the bacterium with pantoprazol (40 mg), amoxicillin (1000 mg) and levofloxacin (500 mg) given for 14 days. Error bars denote SD; *n* = 30 for each group; **P < 0.01, ***P < 0.001 compared with the control.

**Figure 5:**
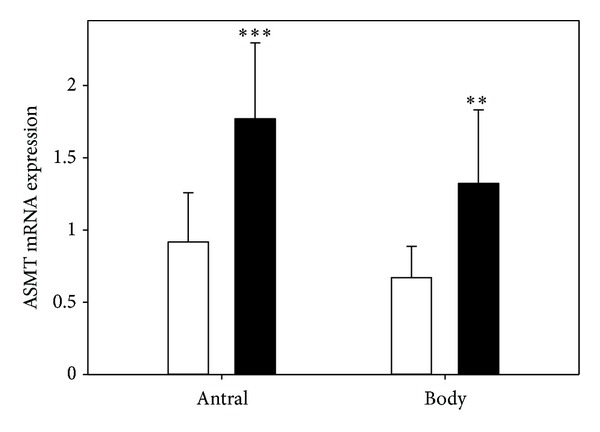
Mean relative level of acetylserotonin methyltransferase (ASMT) expression, measured by real-time PCR, in antral and body gastric mucosa in patients with symptomatic *H. pylori *infection before (control, white bars) and after (black bars) eradication of the bacterium with pantoprazol (40 mg), amoxicillin (1000 mg), and levofloxacin (500 mg) given for 14 days. Error bars denote SD; *n* = 30 for each group; **P < 0.01, ***P < 0.001 compared with the control.

After eradication of *H. pylori,* the expression of AA-NAT (Figure 4) and ASMT (Figure 5) increased in both parts of the stomach. 

In the present study, we observed important differences between the levels of AA-NAT and ASMT expression in gastric mucosa of patients with symptomatic and asymptomatic *H. pylori *infection. The occurrence of dyspeptic symptoms probably does not depend only on the decreased expression of these enzymes but also on other factors and changes in the gastric mucosa. Nevertheless, decreased melatonin secretion, which is certainly suggested by the present findings, may be of significance in the pathogenesis of pain reaction [19, 20]. It was demonstrated that, in *H. pylori* infected patients with functional dyspepsia, melatonin levels in the blood at night were lower than in individuals with asymptomatic infection [21, 22]. In the present study, melatonin secretion was neither investigated during the day nor after *H. pylori* eradication. It seems unlikely, however, that the quantity of melatonin secreted in stomach would affect significantly the concentration of this hormone in blood [2]. An explanation of melatonin's effect on the occurrence of dyspeptic symptoms may lie in its paracrine action. Supporting this hypothesis was the observation that high doses of melatonin inhibited secretion of hydrochloric acid (HCl) and pepsin [23]. However, it should be emphasized that the secretion of HCl itself may not be the cause of pain. Rather, a balance between aggressive (HCl, pepsin) and protective (glycoproteins, bicarbonates) factors is essential in this respect. Melatonin may stimulate the secretion of the mucus, but the mechanism underlying the effect is not clear [24]. A stimulatory effect of melatonin on the secretion of bicarbonates in duodenum and by pancreas glands is more known [25, 26]. This suggests that melatonin may stimulate secretion of bicarbonates also in the stomach. The gastroprotective action of melatonin also may be associated with its antioxidant properties [27–30]. 

Beneficial gastroprotective effects of melatonin in humans have been reported in several studies on experimental animals [31–37]. Both melatonin and its precursor, L-tryptophan, administered in an optimal dose, were shown to accelerate the healing of peptic ulcers and to reduce inflammatory symptoms in upper gastrointestinal tract [38–43]. 

In this work, we observed a negative correlation between the intensity of *H. pylori *infection, measured by the UBT test, and the level of ASMT expression. We could not explain the reason of such correlation neither did we find any report on a relationship between those two quantities. We can only speculate that a cytokine burst associated with an intense *H. pylori* infection may inhibit the expression of ASMT.

*H. pylori *infection is the main cause of peptic ulcers in humans. Colonization by this bacterium evokes inflammatory changes in gastric mucosa, disturbs its structure, and alters functions [44–49]. One of the consequences of the infection is hyperplasia of the gastrin-producing cells (G-cells) with a simultaneous reduction in the number and activity of somatostatin-producing cells (D-cells) in the antral part of the stomach [50–52]. Such changes occur mainly in the first phase of the infection, and they lead to elevated HCl secretion [51–54]. This is likely associated with increase in the number of EC cells [55, 56], but the consequences of this rise are difficult to predict, because these cells secrete both melatonin and serotonin's. Serotonin action is mainly proinflammatory, whereas melatonin has anti-inflammatory properties. Moreover, these effects may be different in the body of the stomach, where the number of EC cells is considerably smaller than in the antral part of this organ. A deficiency of melatonin in gastrointestinal tract may affect not only its secretory function, but also gastric motility, since the indole exerts a myorelaxant effect [57]. It is not known, however, why *H. pylori *colonization causes symptoms only in about 15–20% of the infected individuals. It may depend on the bacterial strain, but it may also be related to the individual's response to the infection, which may involve expression of melatonin synthesizing enzymes [58, 59]. This expression may also change under the influence of the released inflammatory cytokines [60]. Three months after *H. pylori *eradication, the properties of the endocrine cells (EC, ECL, G, D) in the gastric mucosa, as well as their function, were changed. Our results suggest the usefulness of melatonin supplementation in dyspeptic patients, particularly, those infected with *H. pylori *[61]. The use of melatonin as an adjuvant drug for eradication of this bacterium is suggested, but it requires further research. We showed previously that melatonin exerted a beneficial effect in dyspeptic patients not infected with *H. pylori *[62].

Recently, it was shown that melatonin might play an important role in the neurohormonal regulation of duodenal mucosal barrier in rats [63]. If the indole may exert similar effect in human gastric mucosa, it might contribute to the accessibility of *H. pylori *to gastric mucosa cells.

Probably, the most significant weakness of our study was lack of a group without *H. pylori* infection undergoing eradication, but for the ethical reasons, we did not even consider recruiting such group. Therefore, we cannot be sure whether the increase in the expression of AA-NAT and ASMT, observed on the eradication, was the effect of the departing of the bacteria or resulted from the interaction of antibiotics and proton pump inhibitors with the expression system of both enzymes. However, the latter is unlikely, since these compounds exert rather inhibitory not stimulatory general effect. Research on gastric mucosa cells *in vitro* and cell lines might shed some light on this problem, but they do not fit our clinical study.

In conclusion, this study demonstrated that the expression of melatonin synthesizing enzymes, AA-NAT and ASMT, is decreased in the gastric mucosa of individuals with symptomatic *H. pylori* infection, and the use of melatonin as an adjuvant drug in treatment of epigastric pain syndrome is justified.

## Figures and Tables

**Table 1:** Characteristics of the subjects enrolled in the study.

Feature/parameters	Subjects		
	Healthy volunteers	With asymptomatic *H. pylori* infection	With symptomatic *H. pylori* infection
Number of subjects	30	30	30
Age (years)	29.4 ± 8.2	34.6 ± 11.4	30.9 ± 12.6
Gender	F = 13	F = 12	F = 11
	M = 17	M = 18	M = 19
BMI (kg/m^2^)	19.6 ± 0.6	23.6 ± 1.1	21.6 ± 1.4
UBT-13C (‰ CO_2_)	0.9 ± 0.4	23.8 ± 8.3	24.6 ± 16.2
Antral gastritis*	2	29	30
Pangastritis*	—	14	16

*Grade II according to Sydney criteria.
